# Mental Health Effects of Stress over the Life Span of Refugees

**DOI:** 10.3390/jcm7020025

**Published:** 2018-02-06

**Authors:** Michael Hollifield, Teddy D. Warner, Barry Krakow, Joseph Westermeyer

**Affiliations:** 1 The Tibor Rubin VA Medical Center, 5901 East 7th Street, Bldg. 128, Suite 102A, Long Beach, CA 90822, USA; 2 Department of Psychiatry and Human Behavior, The University of California at Irvine School of Medicine, 101 The City Drive, Orange, CA 92868, USA; 3 Department of Family and Community Medicine, University of New Mexico School of Medicine, 2400 Tucker Avenue, Albuquerque, NM 87131, USA; twarner@salud.unm.edu; 4 Sleep and Human Health Institute, 6739 Academy Road, #380, Albuquerque, NM 87109, USA; bkrakow@sleeptreatment.com; 5 Department of Psychiatry and the Minneapolis Veterans Administration Medical Center, University of Minnesota, Minneapolis, MN 55417, USA; weste010@umn.edu

**Keywords:** refugee, trauma, stress, measurement, path models, mental health, post-migration stress

## Abstract

Information about the relative impact of stressful events across the lifespan on the mental health of refugees is needed. Cross-sectional data from a community sample of 135 Kurdish and 117 Vietnamese refugees were fit to a path model about the effects of non-war stress, war-related stress, and post-migration stress on mental health. Kurdish and Vietnamese data were generally consistent with the model. However, war-related stress produced no direct but a large indirect effect through post-migration stress on mental health in Kurds. Vietnamese data indicated a modest direct war-related stress effect but no indirect influence through post-migration stress. Different types of stressful events lead to adverse mental health of displaced refugees in a somewhat group-dependent manner. Implications for prevention and treatment are discussed.

## 1. Introduction

War and other inter- and intra-national conflict has led to the displacement of over 65 million persons worldwide [1]. All of these persons have experienced significant stress, and at least 30% experience distressing emotional symptoms [2], and 10–15% suffer from impairing diagnostic-level psychiatric disorders [3,4,5,6,7,8,9,10,11], the most common being post-traumatic stress disorder (PTSD—10%), major depression (MDD—5%), and generalized anxiety disorder (GAD—4%) with high comorbidity [3]. In some groups, such as Cambodians in Long Beach, CA, USA, disorder prevalence is much higher (62% PTSD, 51% MDD) [8].

This poor mental health of refugees and asylum seekers (hereafter “refugees”) is most commonly attributed to war-related traumatic events. However, early studies show that post-migration stress also contributes to poor mental health in refugees [12,13,14]. Recent work has verified that post-migration stress significantly influences psychopathology in refugees [4,7,10,11,15,16,17]. Furthermore, clinical investigators have opined that problems for refugees in the resettled country are significant and sometimes overwhelm available services [18].

The handful of studies that evaluated the relative contribution of both pre- and post-migration trauma and stress to refugee mental health have found that post-migration stress often provides a risk similar to or greater than war-related trauma [4,8,10,16,17,19,20,21]. Pre- and post-migration stress may differentially predict specific kinds of symptoms and distress in both children and adults [22,23]. However, extant studies do not provide knowledge about direct and indirect effects on health of various types of trauma and/or stress experienced over the lifespan of refugees.

Here we report such effects using path modeling with data from Kurdish and Vietnamese refugees. For simplicity, “stress” and “stressors” are used to indicate types of stressful events that occur over the lifespan, and mental health is the outcome or the reaction to the stressors. Three general stress constructs were evaluated for their relationship to one mental health construct: (1) non-war and non-migration stress (NWS), which theoretically occurred prior to the other two; (2) war-related stress (WRS); and (3) post-migration stress (PMS). Path modeling thus assumed a time-ordered sequence of these stressors. Our hypotheses were that: (1) NWS directly influences WRS and PMS as well as mental health; (2) WRS directly influences both PMS and mental health; and (3) PMS directly influences mental health. The model and hypotheses also imply that the three stress constructs are highly correlated.

## 2. Experimental Section

### 2.1. Study Design

The New Mexico Refugee Project (NMRP) was a two-phase project designed to improve assessment of torture, trauma and health outcomes in refugees. The design and methods of this cross-sectional, retrospective project has been previously described [24]. As part of a larger battery, three instruments were selected a priori for assessing stress across the lifespan of refugees. Three other instruments were selected to evaluate mental health.

### 2.2. Participants and Sampling

Purposive samples were drawn from communities of Kurdish refugees in Colorado Springs, CO, USA and San Diego, CA, USA, and Vietnamese refugees in Albuquerque, NM, USA and have been previously described [24].

The Trauma Experiences Questionnaire (TEQ), developed in our research [5] by partly adapting criteria used by Thompson and McGorry [25], was utilized to sample participants stratified by TEQ category, ethnicity and gender. The TEQ categorized refugees as having experienced either torture, war-related trauma (without torture), or no war-related trauma to obtain a sample having a broad range of experiences related to war and oppression. The United Nations’ Convention against Torture and Other Cruel, Inhuman or Degrading Treatment or Punishment definition of torture [26] was operationalized to identify “torture” survivors. “War-related trauma” survivors experienced war-related events but not torture, and “no war-related trauma” participants had not experienced torture or criterion level other war-related events.

Power analyses determined that a sample size of 240 (3 TEQ groups of 80, substratified by ethnicity and gender) provided power of >0.80 at alpha 0.05 to detect moderate effect sizes between TEQ groups [24]. All participants provided written documentation of informed consent, and were reimbursed $20 for participation. The study was approved by the University of New Mexico’s Institutional Review Board.

### 2.3. Measures and Model Indicators

#### 2.3.1. Instrument Translation and Administration

Translation is complex and must be adapted for specific purposes [27]. All instruments were translated using a rigorous, iterative back-and-forth participatory consensus process with refugees from each language group. This process ensured relevant language-specific semantics and cultural equivalence yielding accuracy and clarity of meaning across groups [24,28]. Instruments were administered to individuals during one session in uniform order as part of the larger battery of assessments.

#### 2.3.2. Stress Constructs and Indicator Measures

During phase I of the NMRP, we developed or adapted instruments to assess the three stressor constructs. Each instrument has multiple inter-correlated sub-scales used as indicators of stress in the path models.

#### 2.3.3. Non-War Non-Migration Stress (NWS) Construct

The *Trauma History Questionnaire* (THQ) is a validated 24-item self-report instrument that assesses: (1) crime-related events; (2) general disaster and trauma; (3) physical and sexual events; and (4) other events [29]. These categories were confirmed by factor analyses in our sample and are indicators of this stress construct in the path models. Test-retest reliability for the 4 composite THQ indicators ranged from *r* = 0.63 to 0.92. Each item is scored either “*not experienced*” or “*experienced*”. The THQ has not been previously used in refugee research, though it has been determined to be the best available instrument to assess NWS [30]. Adaptation of the THQ for this study included translation and alteration of instructions to read, “We are asking you about traumatic experiences you have had that are NOT related to war or migration. Other questionnaires have been about your war and/or migration related experiences: Remember this questionnaire is about traumatic events that are NOT related to war or migration”.

#### 2.3.4. War-Related Stress (WRS) Construct

The *Comprehensive Trauma Inventory-104* (CTI-104) is a reliable and valid self-report that assesses a broad range of war-related traumatic events grouped in 12 sub-scales [24,31]. Respondents check whether or not an event was experienced and, if so, how severe it was in terms of fear or threat to one’s life or safety on a 0–4 scale, designed to assess criterion A of the *Diagnostic and Statistical Manual of Mental Disorders* diagnosis for posttraumatic stress disorder (PTSD). Higher order confirmatory factor analysis indicated that the CTI-104 12 sub-scales could be conceptualized as three WRS subtypes used as indicators in the path model: (1) personal stress; (2) separation and migration stress; and (3) other stress.

#### 2.3.5. Post-Migration Stress (PMS) Construct

The *Post-Migration Living Problems* (PMLP) assesses stressful events during refugee resettlement [13,21,32]. The 23 items are rated on 5-point scales from “no problem” to “a very serious problem”. The PMLP has 5 principal components accounting for 70% of the variance, which were used as indicators of the PMS construct in the path model: (1) refugee determination process; (2) health, welfare and asylum problems; (3) family concerns; (4) general adaptation stressors; and (5) social and cultural isolation. Confirmatory factor analysis with our data supported the validity of these indicators.

#### 2.3.6. Mental Health Construct and Indicator Measures

The *Hopkins Symptom Checklist-25* (HSCL-25) has a 10-item anxiety and a 15-item depression scale, both of which have produced valid and reliable data in the general U.S. population and in various refugee groups [24,30,33,34,35]. An item-average score ≥1.75 for either subscale is clinically significant. Test-retest reliability is high (*r* = 0.89 total, *r* = 0.82 subscales) and predicts diagnosed depression (sensitivity 88%, specificity 73%) or the presence of any major DSM-III Axis I disorder (93% sensitive, 76% specific) in refugees [34].

The *PTSD Symptom Scale-Self Report* (PSS-SR) is strongly associated with war-related experiences in refugees [24], and is a valid predictor of PTSD in U.S. populations [36]. Cronbach’s alpha is 0.91 for total score, and test-retest reliability is 0.74 [36]. The 17 items, each scored 0 to 3 for frequency of symptoms, are DSM-IV PTSD diagnostic symptoms. The PSS-SR may be scored as continuous (1 to 9 = mild; 10 to 19 = moderate; ≥20 = severe), or dichotomous as proxy PTSD diagnosis.

HSCL Depression, HSCL Anxiety, and PSS-SR scores were utilized as indicators of one common mental health construct in the path models.

### 2.4. Data Analyses

Means (*SD*) for the three stress constructs and for the three mental health indicators by ethnicity and gender were calculated. Bivariate correlations between continuous scores for the three stress constructs and the mental health construct were calculated. SPSS Inc., AMOS Version 6.1 (Chicago, IL, USA), was used to fit our proposed structural path model of the effects of the three stress constructs on mental health in Kurdish and Vietnamese refugees.

## 3. Results

### 3.1. Descriptive

#### 3.1.1. Participants

The study included 252 participants: 117 (46%) were Kurdish, 135 (54%) were Vietnamese; 135 (54%) were men and 117 (46%) were women. The mean age was 44 (standard deviation [*SD*] = 14; range 19 to 77), and the Vietnamese were older than the Kurds, *M* = 47 versus *M* = 39 years; *t*(250) = 4.79, *p* < 0.01. On average, Kurds had been in the U.S. for 10 years and the Vietnamese had been in the U.S. for 23 years. On the TEQ, 116 were classified as torture survivors, 84 were survivors of non-torture war trauma, and 48 were refugees who were below criteria for war-related trauma. Four people were not classified by the TEQ. Kurds and Vietnamese were evenly distributed across these recruitment categories. Because sampling was conducted by chain-referral methods, and those who may not have been interested did not contact the research team, true non-participation rates are not available.

#### 3.1.2. Stress Events

Kurds reported more NWS events on the THQ than Vietnamese, *M* = 5.3 (*SD* = 4.1) versus *M* = 3.8 (*SD* = 3.4). The ethnic main effect was *F*(1, 250) = 10.5, *p* < 0.01. Men reported more events than women, *M* = 5.0 (*SD* = 4.0) versus *M* = 4.0 (*SD* = 3.5). The gender main effect was *F*(1, 250) = 4.5, *p* < 0.05.

Kurds also reported more WRS events on the CTI-104 than Vietnamese, *M* = 41.4 (*SD* = 28.5) versus *M* = 23.4 (*SD* = 22.7), *F*(1, 250) = 30.9, *p* < 0.01. Men reported more events than women, *M* = 38.0 (*SD* = 27.2) versus *M* = 24.5 (*SD* = 25.1), *F*(1, 250) = 16.6, *p* < 0.01.

PMS on the PMLP was more pronounced in Kurds than Vietnamese, *M* = 35.9 (*SD* = 23.6) versus *M* = 10.0 (*SD* = 13.5), *F*(1, 250) = 117.9, *p* < 0.01. Table 1 shows the number and percentage of each refugee group that responded either “serious” or “very serious” to each PMLP item, which further demonstrates differences in post-migration stress between groups. PMS was the only stress type that was similar between males and females, *M* = 23.0 (*SD* = 23.8) versus *M* = 20.9 (*SD* = 21.8), *F*(1, 250) = 0.6, *NS*.

#### 3.1.3. Mental Health Status

Kurds had higher mean scores for PTSD, *M* = 15.3 (*SD* = 12.4) versus *M* = 7.0 (*SD* = 8.1), Ethnic main effect *F*(1, 250) = 40.2, *p* < 0.01, depression, *M* = 1.77 (*SD* = 0.67) versus *M* = 1.48 (*SD* = 0.51), *F*(1, 250) = 14.7, *p* < 0.01, and anxiety, *M* = 1.67 (*SD* = 0.63) versus *M* = 1.37 (*SD* = 0.56), *F*(1, 250) = 19.3, *p* < 0.01 than Vietnamese. Furthermore, Kurds were more likely to meet the diagnostic proxy for PTSD on the PSS-SR, 56% versus 24%, *F*(1, 250) = 28.2, *p* < 0.01, and to be above the cut score for clinically significant depression, 41% versus 23%, *F*(1, 250) = 9.8, *p* < 0.01, and anxiety, 34% versus 17%, *F*(1, 250) = 10.2, *p* < 0.01 on the HSCL-25.

Men and women had similar mean scores for PTSD, *M* = 11.7 (*SD* = 11.7) versus *M* = 9.8 (*SD* = 10.4), Gender main effect *F*(1, 250) = 1.8, *NS*, depression, *M* = 1.61 (*SD* = 0.58) versus *M* = 1.62 (*SD* = 0.63), *F*(1, 250) = 0.02, *NS*, and anxiety, *M* = 1.49 (*SD* = 0.58) versus *M* = 1.53 (*SD* = 0.54), *F*(1, 250) = 0.4, *NS*. There were also no gender differences for proxy PTSD diagnosis or percent of respondents above clinically significant cut scores for the HSCL-25 depression and anxiety scales, all *ps* > 0.35.

### 3.2. Correlations among Stress and Mental Health

**Figure 1:**
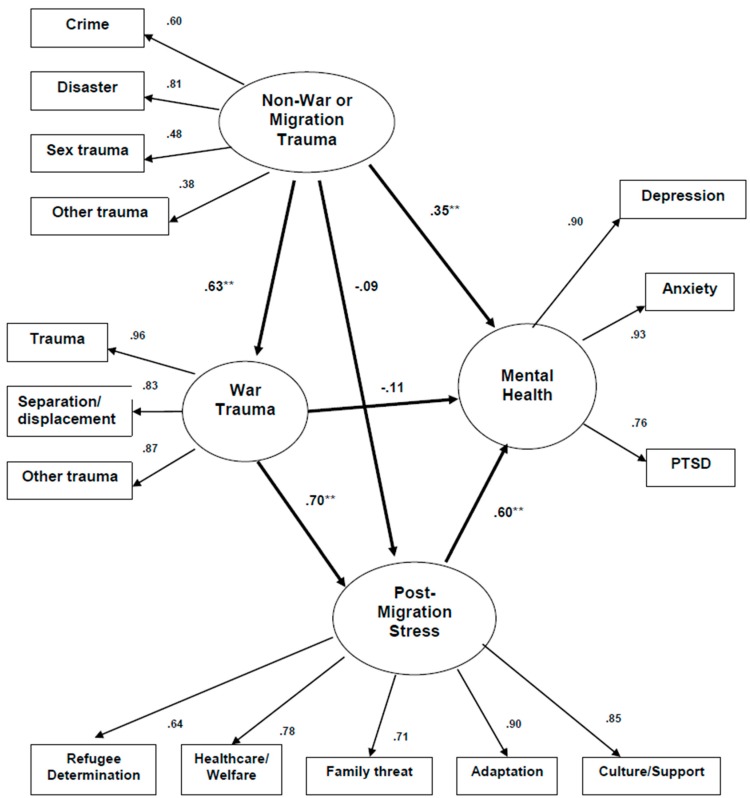
Predicting Mental Health from Various Sources of Trauma: Kurdish (*N* = 117, Standardized Regression Weights and Factor Loadings). ** *P* < 0.01; Full Model: *χ*^2^ = 157.9, *DF* = 84, *p* < 0.001, *RMR* = 1.44, *GFI* = 0.93, *AGFI* = 0.90.

**Figure 2:**
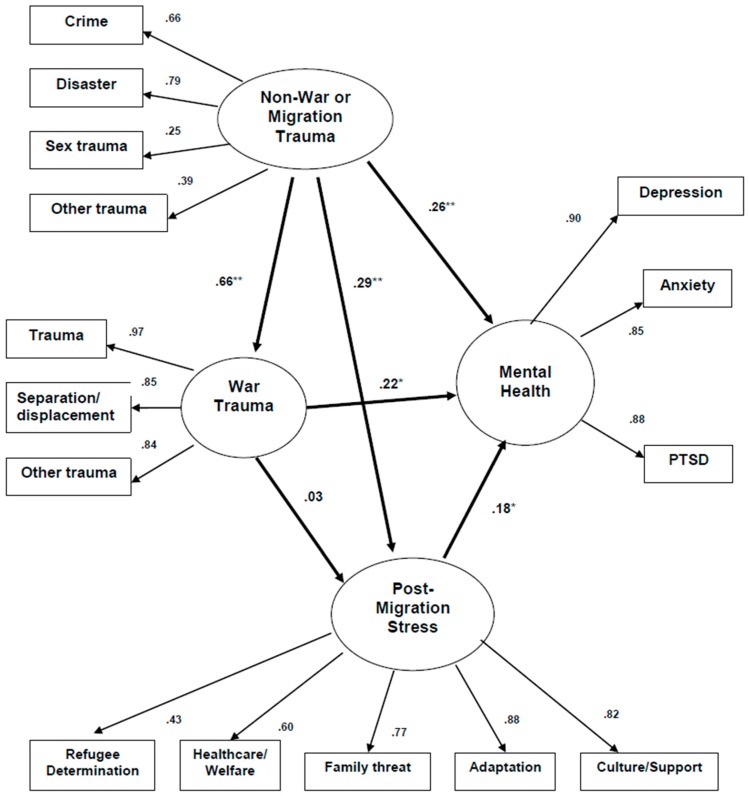
Predicting Mental Health from Various Sources of Trauma: Vietnamese (*N* = 135, Standardized Regression Weights and Factor Loadings). ** *P* < 0.01; * *P* < 0.05; Full Model: *χ*^2^ = 145.1, *DF* = 84, *p* < 0.001, *RMR* = 1.29; *GFI* = 0.93, *AGFI* = 0.90.

As shown in Table 2, the three stress constructs were significantly inter-correlated, all *rs* > 0.31, all *ps* < 0.01. The three mental health indicators were also significantly inter-correlated, all *rs* > 0.72, all *ps* < 0.01. The stress constructs were strongly correlated with the mental health indicators, mean *r* > 0.46 and all *rs* > 0.39, *ps* < 0.01. Correlations of indicators of each of the model’s four constructs are not shown for brevity but are indirectly indicated by the factor loadings displayed in Figure 1 and Figure 2.

### 3.3. Predicting Adverse Mental Health from Stress Across the Lifespan

Factor loadings of the indicators of all four constructs were similar in magnitude across both Kurdish and Vietnamese models, with the exception of the relative strength of association between the PMS construct and the indicators refugee determination process and health, welfare and asylum problems in the Kurds compared to the Vietnamese. This may explain the statistically significant structural paths for the two models being somewhat different.

The overall model for the Kurdish sample fit moderately well, *χ*^2^ = 157.9, *DF* = 84, *p* < 0.001, *RMR* = 1.44, *GFI* = 0.93, *AGFI* = 0.90. However, not all of the predicted paths were statistically significant (Figure 1): (1) NWS showed direct effects on WRS and on mental health, both *p* < 0.01, but no effect on PMS; (2) WRS showed a direct effect on PMS, *p* < 0.01, but surprisingly not on mental health; and, (3) PMS showed a direct effect on mental health, *p* < 0.01.

The overall model for the Vietnamese sample also fit moderately well, *χ*^2^ = 145.1, *DF* = 84, *p* < 0.001, *RMR* = 1.29; *GFI* = 0.93, *AGFI* = 0.90, and a bit better than for the Kurdish sample. In addition, the pattern of significant paths was different than for the Kurdish model (Figure 2): (1) NWS directly influenced WRS, *p* < 0.01, as with the Kurdish model, but also directly influenced PMS, *p* < 0.05, unlike the Kurdish model; (2) NWS did not influence PMS but did directly influence mental health, *p* < 0.05, unlike the Kurdish model; and (3) PMS directly influenced mental health, *p* < 0.05, as with the Kurdish model. At this stage of research, we resisted efforts to refit (overfit) the models to the data, but rely here only on presenting and contrasting the a priori predicted models [37].

## 4. Discussion

This study extends previous research to show that a broad range of stressful events across the lifespan of refugees is related to adverse mental health. Significant differences between refugee groups of direct and indirect effects of stress types on mental health were found. It was not surprising that NWS had both direct and indirect effects on mental health for both groups because these were events that almost always occurred prior to WRS and PMS, and it has been established that prior trauma is a risk factor for adverse effects from later trauma [38]. We hypothesized that WRS would have direct and indirect effects via PMS on mental health, yet there was a direct effect in the Vietnamese and an indirect effect in the Kurds. We believe that PMS as assessed was a much more relevant current stressor for Kurds than for Vietnamese and was thus a more powerful stressor associated with current mental health for Kurds. Most Vietnamese have lived through and had more time to cope with many of the PM stressors assessed, which lessened the effects of PMS in the Vietnamese. These findings emphasize the importance and context dependent variability of various stressors across the lifespan on mental health in refugees. They also highlight the importance of assessment choice. Stress events and cultural and social differences between Kurds and Vietnamese may account for some of the between-group variability in path modeling, but another source of variability may be that Kurds have experienced WRS linked to PMS more recently than the Vietnamese, who are now experiencing stressors that are temporally more distant from their WRS and PMS.

The historical bias toward studying the effects of WRS to the relative exclusion of other kinds of stress in refugees is understandable. War and oppression are the primary events responsible for creating refugees. Images and stories of war trauma, particularly toward the innocent, impress upon us the gruesome and unjust nature of war. The literature is replete with data showing a direct and dose-related effect of WRS to adverse health [8,24,30,39,40]. Models assessing a broader range of stress across the lifespan of refugees have been less examined. One exception is earlier work by Steel and colleagues [21] showing that WRS and PMS both had direct effects on PTSD symptoms and accounted for 20% and 14%, respectively, of the variance in these symptoms. However, Steel and colleagues’ [21] study included one non-representative group of asylum seekers, six main variables but not NWS, and only PTSD as the health outcome.

These and other recent data suggest the need for more research about resettlement and interventions for distressed refugees. Over 6 million of the approximately 65 million globally displaced people suffer from psychiatric disorders [3,41], and severe stress exposure is the most important predictor of mental health status [42]. Refugees also have high rates of medical illness [43,44]. This disease burden is not only due to WRS, although war and oppression are lynchpins for migration and PMS, which is proving to be a significant source of stress and poor health for refugees. The intuition that displacement and resettlement is the best option for oppressed persons has mixed scientific support, even though resettlement is sometimes required due to conditions in the country of origin. Asylum seekers in particular seem to deteriorate psychologically once they are resettled, usually due to imposed restrictions [7,18]. Refugees may show gradual adaptation [45], yet a good comparative study between displaced and re-patriated refugees has not been conducted. The adverse effects of displacement alone on mental health are well documented [21,46]. Refugees lose their place, and along with it status and meaning, identity, and the ability to support their families [47,48]. Countries such as Vietnam and Iraq lose some of their brightest people, who nevertheless struggle in new countries to assert their capacity. The economic cost to individuals and societies is immeasurable. Furthermore, only a minority of resettled refugees with mental disorders access care, and the percentage of internally displaced who are served may be even less. Further study about primary and secondary prevention of stressors and context relevant interventions are needed.

Methodological limitations restrict generalizability of study findings. While the structural model includes a broader range of stress events than in previous work, these events only account for partial variance to adverse mental health. Examples of the contribution of genetic [49], psychological [50], and social [51] variables on poor mental health are well documented. More complete models may shed further light on risk and protective factors for mental health in refugees. Second, the current data are cross-sectional, and models that imply causality, particularly of time-ordered events, would ideally analyze longitudinal data. Furthermore, assumptions about the temporal relationships between the three stress constructs and mental health in the model is unlikely to be fully accurate. Instrument instructions made it clear that WRS precedes PMS, yet not as clear that NWS always occurred before the other two. Instructions provided on the THQ were intended to assess events before war and migration, yet we cannot be certain that this is how each participant interpreted the instructions. Replication studies will benefit from assessment of clear time lapsed epochs by stress type. Third, study data are confined to two ethnic samples that differ in time from stressors and migration. Generalizability will be enhanced by replication from data in similar and dissimilar samples. Finally, PMS as measured by the PMLP may not be as relevant for refugees who have been resettled for a long period of time versus a short period of time (see Table 1 items). Using an assessment that measures current life stressors, to include PMLP items, may be a more realistic construct for modeling. These data nonetheless pose a unique and plausible model of multiple sources of stress on mental health in displaced refugees within a unified analysis that adjusts simultaneously for all variables in the model.

## 5. Conclusions

Multiple sources of stress across the lifespan of war refugees have direct and indirect effects on mental health. Variability in the path of these effects between refugee groups suggests ways in which we might understand the effects of past and ongoing stressors in different populations. Overall path models underscore the imperative to consider policies aimed at prevention and intervention for those harmed by war.

## Figures and Tables

**Table 1:** Post-Migration Stress by Item and by Ethnicity.

Item	Item Response “Serious” or “Very Serious”, Number (%)		Item Score, Mean (*SD*) **	
	Kurds (*n* = 117)	VN (*n* = 135)	Kurds (*n* = 117)	VN (*n* = 135)
Interviews by immigration	17 (15)	9 (7)	0.95 (1.27)	0.47 (0.92)
Conflict with immigration officials	16 (14)	2 (1)	0.80 (1.25)	0.12 (0.52)
No permission to work	14 (12)	1 (1)	0.68 (1.24)	0.07 (0.35)
Fears of being sent home	24 (21)	6 (4)	1.03 (1.50)	0.22 (0.79)
Worries about not getting health treatment	34 (29)	7 (5)	1.44 (1.53)	0.33 (0.82)
Poor access to emergency medical care	37 (32)	6 (4)	1.44 (1.56)	0.35 (0.88)
Poor access to long-term medical care	37 (32)	6 (4)	1.44 (1.53)	0.42 (0.92)
Poor access to dentistry care	45 (38)	11 (8)	1.76 (1.56)	0.60 (1.05)
Poor access to counseling services	31 (26)	11 (8)	1.33 (1.47)	0.56 (1.04)
Little government help with welfare	32 (27)	10 (7)	1.48 (1.59)	0.62 (1.05)
Little help with welfare from charities	29 (25)	11 (8)	1.37 (1.53)	0.54 (1.03)
Delays in processing your application	36 (31)	9 (7)	1.58 (1.53)	0.46 (1.02)
Separation from family	55 (47)	7 (5)	2.03 (1.63)	0.33 (0.86)
Worries about family back at home	78 (67)	15 (11)	2.79 (1.42)	0.84. (1.18)
Unable to return home in emergency	70 (60)	13 (10)	2.57 (1.49)	0.59 (1.12)
Communication difficulties	50 (43)	9 (7)	2.05 (1.53)	0.43 (0.94)
Discrimination	29 (25)	8 (6)	1.40 (1.49)	0.56 (0.95)
Not being able to find work	33 (28)	9 (7)	1.57 (1.49)	0.62 (1.04)
Bad job conditions	35 (30)	4 (3)	1.59 (1.45)	0.36 (0.77)
Poverty	32 (37)	6 (4)	1.53 (1.47)	0.33 (0.87)
Loneliness and boredom	45 (38)	10 (7)	1.92 (1.45)	0.61 (1.07)
Isolation	56 (48)	6 (4)	2.15 (1.52)	0.24 (0.75)
Poor access to the foods you like	16 (14)	4 (3)	0.98 (1.29)	0.35 (0.79)

** *ps* < 0.1 for all item mean scores comparing Ethnic groups.; VN = Vietnamese.

**Table 2:** Correlations among Stress Constructs and Mental Health Indicators.

	Non-War Non-Migration Trauma ^1^	War-Related Trauma ^2^	Post-Migration Stress ^3^	PTSD ^4^	Depression ^5^	Anxiety ^6^
War-related Trauma ^2^	0.57	-	-	-	-	-
Post-migration Stress ^3^	0.31	0.52	-	-	-	-
PTSD ^4^	0.43	0.50	0.54	-	-	-
Depression ^5^	0.39	0.47	0.46	0.74	-	-
Anxiety ^6^	0.41	0.48	0.52	0.72	0.83	-

All correlations significant at *p* < 0.01 (2-tailed); ^1^ Trauma History Questionnaire; ^2^ Comprehensive Trauma Inventory-104; ^3^ Post-migration Living Problems Questionnaire; ^4^ PTSD Symptom Scale-Self Report (PSS-SR); ^5^ Hopkins Symptom Checklist-25 (HSCL-25) Depression Sub-scale; ^6^ Hopkins Symptom Checklist-25 (HSCL-25) Anxiety Sub-scale.
